# User Needs Assessment of ASTHMAXcel Voice: A Novel Mobile Health App for Asthma Management

**DOI:** 10.3233/SHTI250870

**Published:** 2025-08-07

**Authors:** Jamie LEE, Yunan CHEN, Kai ZHENG, Juliana RODRIGUEZ, Jonathan FELDMAN, Sunit P JARIWALA

**Affiliations:** a Department of Informatics, University of California, Irvine, CA, USA; b Albert Einstein College of Medicine, Bronx, NY, USA; c Department of Pediatrics and Department of Psychiatry and Behavioral Sciences, Division of Academic General Pediatrics, Albert Einstein College of Medicine, Montefiore Medical Center, Bronx, NY, USA; d Ferkauf Graduate School of Psychology, Yeshiva University, Bronx, NY, USA; e Division of Allergy/Immunology, Department of Medicine, Albert Einstein College of Medicine and Montefiore Medical Center, Bronx, NY, USA

**Keywords:** Asthma management, mobile health application, digital health, asthma

## Abstract

Existing mobile health apps for asthma predominantly focus on patient education related to self-management. Through a federally-funded project in the U.S., we developed ASTHMAXcel Voice, a novel app that incorporates voice biomarkers and social determinants of health to enhance asthma self-management, shared decision-making, and facilitation of referrals for low-income populations through personalized digital data and collaborations with multiple health services and stakeholders. As part of the user-centered design effort, we conducted a series of user needs assessment focus groups with patients, providers, and community health workers. The results provide valuable insights into (1) perceived usefulness of ASTHMAXcel Voice particularly the new feature utilizing voice biomarkers, and (2) potential adoption and use barriers, and mitigation strategies, when implementing the app in real-world settings.

## 1. Introduction

Asthma disproportionately affects low-income Black and Hispanic residents of the Bronx in New York, where mortality rates are triple that of the state average [1]. Contributing factors include suboptimal self-management skills, environmental exposures, poverty, and other social determinants of health (SDoH) [2]. Effective asthma management requires addressing SDoH and incorporating patient-reported outcomes (PROs) to support self-management and shared decision-making [3]. Yet, the outpatient setting of U.S. healthcare systems faces barriers to collecting, assessing, and responding to SDoH and implementing PROs due to factors such as time constraints and the lack of personnel (e.g. health educators) [4].

Mobile health (mHealth) applications (apps) have the potential to support asthma self-management through features such as guideline-based patient education, personalized management algorithms, push notifications, and provider-facing portals for remote care coordination. Yet, there is a shortage of individualized and rigorously studied mHealth apps to support patient-centered asthma interventions [5]. This research team previously developed and evaluated ASTHMAXcel PRO, an interactive and personalized mHealth app designed to educate and improve self-management in adults with asthma [6]. Building upon this success, we created ASTHMAXcel Voice, a next-generation mHealth app for asthma that incorporates voice biomarkers for early detection of asthma exacerbations, and collection of SDoH data, to further facilitate shared decision-making and referrals to respiratory specialists for low-income populations. This paper reports a user needs assessment study that we conducted while developing ASTHMAXcel Voice. The objectives of the study were twofold: (1) to solicit user feedback of the app particularly its innovative feature utilizing voice biomarkers, and (2) to identify and understand potential barriers to the adoption and long-term use of the app when it is deployed in real-world settings.

## 2. Methods

### 2.1. Novel features of the ASTHMAXcel Voice app

ASTHMAXcel Voice incorporates a voice-based asthma exacerbation early detection feature that is based on a voice biomarker platform developed by Sonde Health (Sonde Health, Inc., Boston, Massachusetts, U.S.). The feature assesses the patient’s respiratory dysfunction from a 6-second voice sample using machine learning techniques. The resultant asthma risk score is combined with the SDoH data collected within the app to provide more accurate exacerbation assessments in addition to shared decision-making capabilities between patients and providers. As such, ASTHMAXcel Voice takes a holistic patient-centered approach to asthma management by integrating personalized digital data (e.g., SDoH, PROs, voice biomarkers) and supporting the collaboration between patients and various health services and stakeholders.

### 2.2. Research design

This study took place between August 2023 and January 2024. Participants from three key stakeholder groups: patients, healthcare providers, and community health workers (CHWs), were recruited from multiple outpatient primary and specialty care sites in Bronx, New York. A total of 7 focus group sessions were conducted with 36 participants in total. Among them, 5 focus groups were conducted with patients (n=19), 1 with healthcare providers (n=8), and 1 with CHWs (n=9). Each session was led by two researchers and lasted approximately 30 minutes.

The focus group protocol includes questions soliciting feedback from patient participants on how to best refine ASTHMAXcel Voice to their needs and from providers and CHWs on how they can use the app to facilitate asthma screenings and referrals. All sessions were voice-recorded and then transcribed. The theoretical framework guiding the qualitative analyses of the transcripts is the classic Technology Acceptance Model [7] which posits that prospective users’ decision to reject or embrace a new technology is determined upon several behavioral antecedents such as perceived usefulness and perceived ease of use.

## 3. Results

### 3.1. High perceived usefulness of voice-based asthma risk scores

Participants of all types perceived the voice biomarker feature to be useful in helping with asthma management. Patients believed that they could learn more about asthma symptoms and triggers by viewing the voice-based asthma risk scores. For example, such scores could *“be helpful because sometimes you may think you’re having an asthma attack when you’re not having it.”* (Group 2, Patient 2). The overall feedback from providers and CHWs was also positive with high levels of perceived usefulness of the new feature. They believed that the voice-based risk scores would not only inform patients of their triggers, as *“it’s very helpful for someone to know what are the triggers”* (Group 2, Provider 1), but also inform the providers as they *“would like to see just three or four major factors that influence [the patient’s] asthma”* (Group 1, Provider 1). They also felt that utilizing mobile apps could be more advantageous as teenagers *“are more savvy with their phones rather than doing the peak flow... So, I think [ASTHMAXcel Voice] should work out well for all”* (Group 1, Provider 1).

Each group offered concrete suggestions on how to better present data in the app. Patients felt that the asthma score generated from the voice sample should be displayed as a numerical number with additional, easy-to-understand information. More specifically, they wanted to see a descriptive label associated with the score (e.g., very bad, bad, moderate, good, very good) and factors that could be triggering the asthma risk score to fail into a particular range. They also wanted the ability to view a daily log of the scores to analyze triggers over time. Many patients expressed willingness to use the app on a daily basis, suggesting that custom reminder notifications would be beneficial as *“sometimes you have so much going on in your every day life that you tend to forget these things”* (Group 4, Patient 3). Additionally, patients wanted guidance on actionable steps after receiving the score. One patient suggested that the app should *“give tips and tricks that would help you with managing your breathing depending on the score”* (Group 2, Patient 2).

The providers and CHWs participating in the study expressed a desire to have access to additional data alongside the asthma risk scores such as medication usage and environmental triggers. For example, one provider stated: *“*... *at the very least if they’re not using [controllers] enough that we should get that information”* (Group 1, Provider 3). With additional data on patients’ use of asthma controllers, providers can at least *“tweak their medications here and there”* since they *“cannot go to [the patients’] house and change their living environment in whatever way”* (Group 1, Provider 1). On the other hand, some providers and CHWs raised questions about the voice biomarker feature particularly on the accuracy of voice-based assessment. One participant asked: *“are there any confounding factors that can affect the score without it being asthma deterioration?”* (Group 1, Provider 5) Additionally, the same participant questioned how the mobile app would *“compare with the old lower tech monitoring program where the patient will just blow a peak flow meter...?”* (Group 1, Provider 5).

### 3.2. Questions regarding the feasibility of implementation in clinical settings

Most concerns regarding the new app were from providers and CHWs, focusing on the feasibility of its implementation on the clinical side. First, it was not clear what data will be shown to providers, how they would access the data, and how they should use the data. For example, after the interviewer had explained about how patients receive the asthma risk score, a CHW asked, *“I like that but what comes after that? Like the doctor will follow up with them?”* (Group 2, CHW 4). Another provider asked, *“How does clinical care get affected once [the patients] start using [the app]?”* (Group 1, Provider 1). Further, providers and CHWs were uncertain about their roles in supporting patients’ asthma self-management while using the app, including what the next actionable step was. A provider asked, *“Let’s say a patient ignores the score that they have. What happens next?”* (Group 1, Provider 3). The workflow for data collection and integration was also ambiguous to some providers and CHWs. It was unclear how the SDoH data would be collected to facilitate referrals to CHWs. A CHW inquired, *“How does [the app] connect back to a community health worker?*...*For [patients] to get a referral for someone to assist them”* (Group 2, CHW 3).

While discussing these concerns, providers and CHWs also provided suggestions on how to best implement the app to work with the established clinical workflow and referral processes. When asked about integrating asthma data, for example, providers generally agreed that Epic, a widely used electronic health record (EHR) system, “*would be the best way to [transmit information]*” (Group 1, Provider 3). This provider further commented that it would be helpful to have some notes or alerts in Epic indicating the patient is participating in the study. More concrete suggestions about where exactly such notes or alerts should be placed in Epic were made: “a tab on the side” of the implant information, or “a link to the Flow Sheet.” As for facilitation of the referral process, providers and CHWs wanted a quick summary to show if a patient qualifies for certain programs (e.g., Healthy Homes). Another CHW asked if trigger data could “*be sent to Epic so that the provider can automatically say, hey, this person is a referral to a community health worker?*” (Group 2, CHW 2), highlighting again the importance for EHR integration. Another participant noted that “*most of the time when [CHWs] are addressing housing quality issues, some of the patients don’t follow through the steps they have to take in order to resolve the issues,*” suggesting providers should reach out to patients and encourage them to “*really follow the steps so that we can help address this issue*” (Group 2, CHW 2).

## 4. Discussion

In this study, we conducted user needs assessment focus groups with patients, providers, and CHWs to gather feedback on developing the ASTHMAXcel Voice app that incorporates novel features such as voice biomarkers and SDoH data capture. The findings highlight the potential benefits of the new features in better supporting asthma self-management, shared decision-making, and facilitation of referrals. They also point to concerns regarding the feasibility of introducing the app in clinical settings and the critical need to integrate it with existing clinical workflow and health IT systems.

For patients, while the voice-based asthma risk scores are believed to provide critical information about their current condition and triggers, this data must be presented in ways that are easily understood by the target population as lower health literacy has been associated with poorer asthma outcomes [8]. Data-driven technologies are often not inclusively designed for individuals with complex data needs or lower literacy skills, such as young children [9]. Several strategies include employing simple charts and graphs, avoiding medical jargon, and providing multi-language options.

Lastly, the findings underscore the importance of providing tangible benefits to medical professionals when implementing new, consumer-facing health technologies and integrating them seamlessly into existing clinical workflow to ensure usability and effectiveness. Further, while SDoH are increasingly collected in clinical settings, referrals for support services may occur in fewer than 20% of patients with a positive screen [10]. Therefore, implementing systematic steps to efficiently integrate SDoH into clinical decision-making is essential to ensure providers and CHWs have accessible and actionable data to facilitate referrals.

## 5. Conclusion

This study used focus groups to explore patient, provider, and CHW reactions to ASTHMAXcel Voice, a new-generation mHealth app for asthma management. The results highlight the need to present health data in clear ways, such as through intuitive visualizations and accessible language, to aid patient understanding of symptoms, triggers, and asthma status. On the clinical side, our findings stress the need to bridge the gap between patient asthma data and clinical workflow, particularly ensuring seamless data integration into existing EHR systems. These insights lay the foundation for iterative development and broader implementation of patient-centered, workflow-compatible digital health tools such as ASTHMAXcel Voice for asthma management.
